# Disgust as an emotional driver of vaccine attitudes and uptake? A mediation analysis

**DOI:** 10.1017/S0950268819000517

**Published:** 2019-04-26

**Authors:** P. M. Luz, H. E. Brown, C. J. Struchiner

**Affiliations:** 1 Instituto Nacional de Infectologia Evandro Chagas, Fundacao Oswaldo Cruz, Av. Brasil 4365, Manguinhos, Rio de Janeiro, Brazil; 2 Department of Epidemiology and Biostatistics, Mel and Enid Zuckerman College of Public Health, 1295 N Martin Ave, University of Arizona, Tucson, AZ, USA; 3 Escola de Matemática Aplicada, Fundação Getúlio Vargas, Praia de Botafogo, 190, Rio de Janeiro, Brazil

**Keywords:** Barriers, disgust, influenza, susceptibility, vaccine attitudes, vaccine uptake

## Abstract

Research on the drivers of vaccine acceptance has expanded but most interventions fall short of coverage targets. We explored whether vaccine uptake is driven directly or indirectly by disgust with attitudes towards vaccines acting as a possible mediator. An online cross-sectional study of 1007 adults of the USA via Amazon's Mechanical Turk was conducted in January 2017. The questionnaire consisted of four sections: (1) items assessing attitudes towards vaccines and vaccine uptake, (2) revised Disgust Scale (DS-R) to measure Disgust Sensitivity, (3) Perceived Vulnerability to Disease scale (PVD) to measure Germ Aversion and Perceived Susceptibility, and (4) socio-demographic information. Using mediation analysis, we assess the direct, the indirect (through Vaccine Attitudes) and the total effect of Disgust Sensitivity, Germ Aversion and Perceived Susceptibility on 2016 self-reported flu vaccine uptake. Mediation analysis showed the effect of Disgust Sensitivity and Germ Aversion on vaccine uptake to be twofold: a direct positive effect on vaccine uptake and an indirect negative effect through Vaccine Attitudes. In contrast, Perceived Susceptibility was found to have only a direct positive effect on vaccine uptake. Nonetheless, these effects were attenuated and small compared to economic, logistic and psychological determinants of vaccine uptake.

## Introduction

Despite vaccines being the most important public health intervention to date [1], some individuals and/or communities choose not to vaccinate, mostly for personal (religious and philosophical) reasons. Recent studies have demonstrated that communities with low vaccine coverage may experience outbreaks with high case-fatality rates [2]. To address this, research on the drivers of vaccine acceptance has greatly expanded, from the theoretical framework to the individual, social and structural factors that influence it [3–7]. For strictly pro- or anti-vaccine groups, the decision to get vaccinated is likely straight-forward leading them to vaccinate or not, respectively. However, the group that hesitates with regard to vaccines likely includes those who are unsure of their assessment of vaccines (which may well be vaccine-specific), as well as those who have no knowledge, no interest or no time. The relatively small effect of most interventions (with the exception of mandatory vaccination [8]) aimed at decreasing anti-vaccine attitudes, increasing vaccine knowledge and/or promoting vaccine uptake [9] suggests that the link between attitudes, beliefs, knowledge and behaviour is multifaceted. In fact, approaches aimed at correcting vaccination misinformation among vaccine sceptics have been shown to have no effect or even backfire [10–12] while employers that mandate vaccination may face litigation [13].

Reluctance to vaccines may also result from one's attitudes towards vaccines being driven by emotional states more so than cognitively acquired knowledge. In particular, the emotion of disgust stands out as a possible underlying emotion driving attitudes and/or behaviours. Disgust is a basic emotion, experienced as a transitory emotional state that evolved to motivate contamination avoidance [14]. Disgust has been characterised as a ‘behavioural immune system’ since, like our biological immune system that fights off infection, our behavioural immune system motivates avoidance of people or situations that might result in contamination [15]. The choice to vaccinate requires rational decision-making process but, if disgust drives vaccine attitudes, as a basic emotion, it may be difficult to cognitively override [16, 17]. Indeed, disgust's possible role in shaping vaccine attitudes could partially explain the difficulty in correcting misperceptions about vaccination [10–12, 18].

Studies have shown people vary in their tendency (propensity and sensitiveness) to experience disgust in response to potential elicitors, a characteristic usually denominated ‘disgust sensitivity’ [14]. To date, no study has explored the possible link between disgust and the manifest behaviour, vaccine uptake, though three studies have assessed its association with a person's beliefs and attitudes towards vaccines [19–21]. Disgust may influence attitudes towards vaccines with two opposing hypotheses on the directionality of the association. Since vaccines prevent infection, those with heightened disgust sensitivity might hold more positive attitudes towards vaccines so as to prevent themselves from infection. In contrast, those with heightened disgust sensitivity could hold more negative attitudes towards vaccines perhaps because they view the vaccines themselves as contaminants. These three studies found that participants with heightened disgust sensitivity held more negative attitudes towards vaccines [19–21]. These studies also showed that participants with anti-vaccination attitudes were prone to conspiracy thinking, had low tolerance of limits to their freedom and more strongly opposed genetically modified foods, among other characteristics. Furthermore, vaccine conspiracy beliefs have been shown to be negatively associated with parents’ willingness to vaccinate their sons with the HPV vaccine [22].

In the present study, we expand prior research using validated instruments by testing whether the constructs, Disgust Sensitivity, Germ Aversion and Perceived Susceptibility, measured by these instruments are linked to vaccine attitudes and/or behaviour. Using mediation analysis, we estimate direct and indirect effects, the latter through Vaccine Attitudes, of the constructs on the manifest behaviour, self-reported vaccine uptake, measured at the closing of the flu vaccine season. Next, we evaluate the strength of these effects when controlling for factors previously found to predict flu vaccine uptake [23].

## Methods

### Study design and population

Data from 1007 Amazon's Mechanical Turk (MTurk) participants were collected in January 2017 for this cross-sectional study. By the end of December 2016, approximately 143.7 million doses (98% of the 145.9 million doses given that season) had been administered [24]. After reading a brief study description, a link was provided leading potential participants to the Qualtrics platform. Subsequent to obtaining electronic informed consent, participants were directed to the survey questions. Eligibility criteria (established within MTurk's platform) was minimum age of 18 years and USA residence. Once started, the survey had to be completed within 1 h (expected average time 10 min). The survey remained available until the maximum number of participants (1000) was reached. Seven additional surveys were accepted because of our error in closing the survey. No exclusion criteria were applied either before or after data collection. Participants were compensated for their time based on a recommended pay rate and the average time for questionnaire completion [25].

### Instrument and measures

The questionnaire consisted of four sections: (1) items used to measure attitudes towards vaccines and vaccine uptake, (2) the revised version of the Disgust Scale (DS-R), (3) the Perceived Vulnerability to Disease scale (PVD) and (4) socio-demographic information. To minimise question order effect bias, the first three sections were presented to participants at random. The fourth and always final section included eight items collecting the individual's socio-demographic information.

From section 1, attitudes towards vaccines and vaccine uptake, seven items were used in this analysis. One inquired on individual's vaccine behaviour in the fall of 2016 (‘Were you vaccinated against the flu this past season (fall of 2016): yes/no’). Six items assessed expressed general attitudes towards vaccines’ efficacy and safety, e.g. ‘People get sick from vaccines’ (Table 1). Comparing our items with the ‘3 Cs’ model proposed by the SAGE Working Group, we see that our Vaccine Attitudes items address the Confidence category defined as ‘trust in the effectiveness and safety of vaccines; the system that delivers them, including the reliability and competence of the health services and health professionals and the motivations of policy-makers who decide on the needed vaccines’ [26]. Items were measured on a five-point Likert-type scale ranging from strongly agree (scored as −2) to strongly disagree (scored as +2) for the four items expressing negative positions towards vaccines while the other two items were reverse scored (‘Vaccinating healthy young children helps protect others by stopping the spread of disease’ and ‘Doctors would not recommend vaccines if they were unsafe’) such that higher values indicate a more positive attitude towards vaccines.

**Table 1.** Six-item Vaccine Attitudes scale intended to measure people's general attitudes towards vaccines, bivariate correlations and factor loadings

		Bivariate correlations						Factor loading
	*n* = 1007	1	2	3	4	5	6	
1	People get sick from vaccines	1.00	0.53	0.45	0.48	0.53	0.58	0.69
2	The risk of side effects outweighs any protective benefits of vaccines		1.00	0.47	0.51	0.45	0.52	0.62
3	Vaccinating healthy young children helps protect others by stopping the spread of disease – R			1.00	0.67	0.55	0.57	0.68
4	Children do not need vaccines for diseases that are not common anymore				1.00	0.47	0.65	0.76
5	Doctors would not recommend vaccines if they were unsafe – R					1.00	0.58	0.68
6	Vaccines weaken the immune system						1.00	0.80

R, indicates items that were reverse scored.

The revised version of the DS-R [27] is a 25-item self-report scale that measures Disgust Sensitivity. An individual's Disgust Sensitivity score was computed as per instrument's instructions concerning reverse coding of items, scoring of the responses, as well as the computation of the total score which was calculated by summing responses to the 25 items (range 0–25); higher scores indicate greater disgust sensitivity. The DS-R has strong psychometric properties [27] and, in the present sample, Cronbach's *α* estimate for the Disgust Sensitivity total score was acceptable (0.85) with an average interitem correlation of 0.20 (range 0.01–0.46).

The PVD [28] is a 15-item self-report scale that measures two conceptually distinct constructs: Germ Aversion (or contamination disgust, eight items, e.g. ‘I prefer to wash my hands pretty soon after shaking someone's hand’), and Perceived Infectability (seven items, e.g. ‘In general, I am very susceptible to colds, flu and other infectious diseases’). Herein, we renamed the Perceived Infectability construct, defined as an ‘individuals’ beliefs pertaining to their susceptibility to infectious diseases’ [28] to Perceived Susceptibility, a term more commonly used in the medical/public health literature [29]). Subscales scores were computed (according to instrument's instructions) as the mean of all items within a factor after being scored on a seven-point Likert-type scale ranging from strongly agree (scored as 1) to strongly disagree (scored as 7): higher scores indicate greater Germ Aversion (range 1–8) and Perceived Susceptibility (range 1–7). The internal consistency of the two subscales was adequate and acceptable in the present sample: Cronbach's *α* 0.90 for Perceived Susceptibility and 0.78 for Germ Aversion.

### Analysis

Initially, we assessed the appropriateness of the six items thought to measure Vaccine Attitudes with correlations and principal axis factor analysis. Subsequently, internal consistency was assessed with Cronbach's *α* which was deemed questionable if >0.60 and <0.70, acceptable if >0.70 and <0.80, and adequate if >0.80. An individual's Vaccine Attitudes score was then computed as the average of the items. Bivariate correlations between Vaccine Attitudes, Disgust Sensitivity, Germ Aversion and Perceived Susceptibility were assessed using Spearman's correlation coefficient. Differences in Vaccine Attitudes, Disgust Sensitivity, Germ Aversion and Perceived Susceptibility were assessed for demographic variables and tested using Kruskal–Wallis test.

Subsequently, we assessed the role of Disgust Sensitivity, Germ Aversion and Perceived Susceptibility on flu vaccine uptake, the binomial outcome of the logistic regression model. Using mediation analysis, we explored whether the association between the exposures (Disgust Sensitivity, Germ Aversion and Perceived Susceptibility) and the outcome (flu vaccine uptake) results from a direct effect or an indirect effect through a mediator (Vaccine Attitudes).

In recent years, causal mechanisms have been studied within the modern framework of causal inference. Under this framework, we define direct and indirect effects, the quantities of interest in a causal mediation analysis, by comparing the observed and potential outcomes of the concept of interest (flu vaccine uptake) under different combinations of treatment/exposure (either Disgust Sensitivity, Germ Aversion or Perceived Susceptibility) and the intermediate/mediator variable (Vaccine Attitudes). Potential outcomes are not directly observed and require identification via statistical models along with specific assumptions. The statistical theory that underlies the procedures used in our analyses are described in the literature [30, 31], and implemented, respectively, in the ‘mediation’ [32] and ‘medflex’ [33] packages in R (The R Project, https://www.r-project.org/). We used both packages for our analysis and obtained the same results, we report the estimates obtained with package ‘medflex’.

Finally, we ran logistic regression models while accounting for factors previously identified as significant predictors of vaccine uptake [23]: (1) annual income, (2) access to the flu vaccine at workplace, (3) belonging to a priority group for which the flu vaccine is highly recommended by the Centers of Disease Control and Prevention (CDC), (4) perceived susceptibility to flu (e.g. ‘My chances of getting the flu are high’), (5) perceived risk of infection without the vaccine, (6) perceived benefits of the flu vaccine (e.g. ‘Getting a flu shot will prevent me from getting the flu’, (7) perceived barriers to uptake the flu vaccine (e.g. ‘There are too many risks in getting a flu shot’).

### Ethical consideration

The study protocol was reviewed by the University of Arizona Institutional Review Board and deemed exempt.

## Results

### Sample characteristics

A total of 1007 participants answered the survey (taking on average 9 min), most were aged 25–44 years: 674/66.9% (18–24 years: 106/10.5%, 45+: 227/22.6%), 50% were male (503), 40.3% (404) reported some college or less (59.7%/598 reported associate/bachelor/graduate degrees) and 61% (614) reported an annual income ⩾30 000 and <100 000 (<30 000: 270/26.8%, >100 000: 123/12.2%) (Table 2). Flu vaccine uptake in the fall of 2016 was reported by 317 (31.5%).

**Table 2.** Socio-demographic characteristics of the study population

Total	1007
Age (years)	
18–24	106 (10.5)
25–44	674 (66.9)
45+	227 (22.6)
Male sex	503 (50)
Education	
Some college or less	404 (40.3)
Associate/bachelor/graduate degrees	598 (59.7)
Annual household income (US$ thousands)	
<30	270 (26.8)
⩾30 and <100	614 (61)
>100	123 (12.2)

Bivariate correlations between the six items thought to measure Vaccine Attitudes was high (0.45–0.67, Table 1). Principal axis factor analysis showed that the six items converged into one factor with loadings varying from 0.80 for item 6 to 0.62 for item 2; internal consistency was adequate: Cronbach's *α* = 0.85 (Table 3). Accordingly, Vaccine Attitudes scores were computed as the mean of the six items (median 0.83, interquartile range (IQR) 0.17–1.5, mean 0.80, standard deviation 0.88, range −2 to +2). Median Vaccine Attitudes score did not significantly differ by age group (*P* = 0.54), sex (*P* = 0.68) or income bracket (*P* = 0.20) but those with higher education reported more positive attitude towards vaccines (median Vaccine Attitudes score of 0.7 for those with some college or less and 1.0 for associate/bachelor/graduate degrees, *P* < 0.001).

**Table 3.** Mean, standard deviation, Cronbach's *α* and bivariate correlations

*n* = 1007	1	2	3	4	Mean	s.d.
1. Vaccine attitudes	1	−0.18*	−0.18*	−0.02	0.80	0.88
2. DS-R disgust sensitivity		1	0.56*	0.21*	14.3	4.84
3. PVD germ aversion			1	0.28*	4.3	1.09
4. PVD perceived susceptibility				1	3.2	1.18
Cronbach's *α*	0.83	0.85	0.78	0.90		

DS-R, Disgust Sensitivity scale – Revised version; PVD, Perceived Vulnerability to Disease scale; s.d., standard deviation.

There were no missing data.

*Indicates significant correlation at the *P* = 0.05 threshold.

### Disgust sensitivity, germ aversion and perceived susceptibility

Disgust Sensitivity (mean score 14.3, median 14.5, IQR 11–18, range 1.5–25), as reported previously [27], was significantly higher among women (DS-R: 16.0 women, 13.0 men, *P* < 0.001) and younger individuals (DS-R: 15 for ⩽65 years, 11 for >65 years old, *P* = 0.04). For the PVD subscales, overall scores were mean of 4.3 (median 4.27, IQR 3.5–5, range 1.1–7) for Germ Aversion and 3.2 (median 3.1, IQR 2.4–4, range 1–7) for Perceived Susceptibility. Women scored higher in both PVD subscales (Germ Aversion: 4.5 women, 4.0 men; Perceived Susceptibility: 3.4 women and 3.0 men, both *P* < 0.001), as reported previously [28]. Vaccine Attitudes scores correlated negatively and significantly with Disgust Sensitivity and Germ Aversion (both *P* < 0.0001) but not with Perceived Susceptibility (*P* = 0.58) (Table 3). Vaccine Attitudes scores were found to differ significantly among those who chose to vaccinate in the fall of 2016: median score of 0.8 (IQR 0–1.3) among the 690 who did not vaccinate *vs.* 1.2 (IQR 0.5–1.5) among the 317 who did vaccinate (*P* < 0.001).

### Mediation analysis

**Fig. 1.**
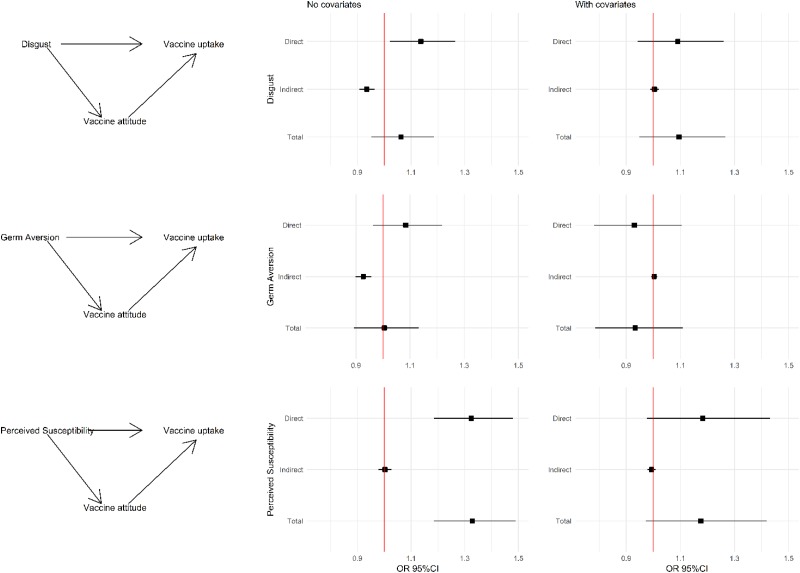
Mediation analysis: left column: causal diagrams showing the direct and indirect (through vaccine attitude) effects of disgust sensitivity, germ aversion and perceived susceptibility on vaccine uptake. Middle column: odds ratio and 95% confidence interval for the estimated direct, indirect and total effects. Right column: odds ratio and 95% confidence interval for the estimated direct, indirect and total effects when also adding covariates to the model. *Note*: Disgust score was adjusted to the same scale as the other variables by dividing the score by four. Footnote: Covariates included in the model were: annual income, vaccine offered at the workplace, belong to a priority group as per CDC recommendations, perceived susceptibility to flu, perceived risk of infection without vaccination and perceived benefits and barriers of the flu vaccine.

Mediation analysis showed that the effect of Disgust Sensitivity on vaccine uptake was twofold: there was a direct positive effect of Disgust Sensitivity on vaccine uptake (Table 4 and Fig. 1, odds ratio (OR) 1.14, 95% CI 1.02–1.27) and an indirect negative effect through Vaccine Attitudes (OR 0.93, 95% CI 0.91–0.96). Given their opposing directions, the ‘apparent’ total effect of Disgust Sensitivity on vaccine uptake is null (OR 1.06, 95% CI 0.95–1.18). The result was similar for Germ Aversion: a direct positive effect (OR 1.08, 95% CI 0.96–1.22) and indirect negative effect (OR 0.92, 95% CI 0.90–0.96). Again, the total effect is null (OR 1.01, 95% CI 0.89–1.13). Perceived Susceptibility exhibited a very different pattern, with a stronger positive direct effect (OR 1.32, 95% CI 1.19–1.48) and a null indirect effect. As such, its total effect (OR 1.33, 95% CI 1.18–1.49) was composed of its direct effect only.

**Table 4.** Estimates of direct and indirect causal effects, and corresponding confidence intervals, of Disgust Sensitivity, Germ Aversion and Perceived Susceptibility on vaccine uptake obtained by fitting a logistic regression model with and without covariates to the expanded dataset representing counterfactual outcomes

	Disgust Sensitivity	Germ Aversion	Perceived Susceptibility
No covariates			
Direct	1.14 (1.02–1.27)	1.08 (0.96–1.22)	1.32 (1.19–1.48)
Indirect	0.93 (0.91–0.96)	0.93 (0.90–0.96)	1.00 (0.98–1.03)
Total	1.06 (0.95–1.18)	1.01 (0.89–1.13)	1.33 (1.18–1.49)
With covariates			
Direct	1.09 (0.94–1.26)	0.93 (0.78–1.11)	1.18 (0.98–1.43)
Indirect	1.00 (0.99–1.02)	1.00 (0.99–1.01)	0.99 (0.98–1.01)
Total	1.10 (0.95–1.27)	0.93 (0.79–1.11)	1.18 (0.97–1.42)
Covariates			
Annual income $30–99th. (Ref. <30)	2.01 (1.30–3.09)	2.06 (1.33–3.17)	1.99 (1.30–3.05)
Annual income $100th.+ (Ref. <30)	1.76 (0.94–3.30)	1.81 (0.96–3.40)	1.74 (0.93–3.25)
Vaccine offered at workplace (*vs*. not)	3.48 (2.36–5.13)	3.44 (2.34–5.08)	3.59 (2.42–5.31)
Belong to priority group (*vs*. not)	3.82 (2.49–5.87)	3.79 (2.46–5.84)	3.76 (2.44–5.79)
Perceived susceptibility to flu infection	2.27 (1.79–2.87)	2.41 (1.89–3.06)	2.09 (1.59–2.73)
Perceived risk without vaccine	1.26 (1.03–1.55)	1.27 (1.04–1.56)	1.27 (1.03–1.56)
Perceived benefits of flu vaccine	1.99 (1.63–2.43)	1.99 (1.63–2.42)	2.01 (1.65–2.45)
Perceived barriers of flu vaccine	0.29 (0.23–0.38)	0.30 (0.24–0.39)	0.29 (0.23–0.38)

Prior logistic regression model (adjusted odds ratio, 95% confidence interval): annual income $30–99th. (2.03, 1.32–3.11), annual income $100th.+ (1.79, 0.98–3.25), vaccine offered at workplace (3.45, 2.34–5.07), belonging to a priority group (3.80, 2.50–5.78), perceived susceptibility to flu infection (2.34, 1.86–2.94), perceived risk without vaccine (1.27, 1.02–1.57), perceived benefits of vaccine (1.99, 1.63–2.44) and perceived barriers of flu vaccine (0.30, 0.24–0.38).

We then tested these models while including variables we previously found to predict vaccine uptake [23]. The direct and indirect effects of Disgust Sensitivity and Germ Aversion were no longer significant. In contrast, the direct positive effect of Perceived Susceptibility remained relatively strong and borderline significant (adjusted OR 1.19, 95% CI 0.98–1.43). Adding Disgust Sensitivity, Germ Aversion or Perceived Susceptibility to the prior adjusted model had a negligible impact on the effect size of the described predictors. Moreover, only Perceived Susceptibility remains borderline significant in the adjusted model where it leads to a reduction of the effect size of perceived susceptibility to flu infection.

## Discussion

We show two opposing roles for disgust on vaccine uptake, a direct positive effect combined with an indirect negative effect through Vaccine Attitudes, which in-turn influences the manifest behaviour in the absence of other predictors. Additionally, we found that those who perceived themselves as more susceptible to infection were more likely to be vaccinated, while no indirect effect of Perceived Susceptibility through Vaccine Attitudes was observed. Nonetheless, these effects were small and attenuated by economic, logistic and psychological determinants shown in prior analyses to be associated with vaccine uptake [23]. In particular, the psychological constructs of perceived susceptibility to flu and perceived benefits of flu vaccine more than doubled the odds of flu vaccine uptake while the more generic assessment of Perceived Susceptibility had only a minor effect size. Moreover, exceeding the effect sizes of perceived susceptibility to flu and perceived benefits of flu vaccine, factual (economic and logistic) and perceived barriers to getting the flu vaccine were the strongest predictors of flu vaccine uptake. Our inclusion of these variables in the models considered only their independent effect on the outcome, future studies should explore alternative conceptual models. Furthermore, our conceptual model assumed that a person's general attitudes towards vaccines led to their behaviour during the 2016 flu vaccine season yet it is also plausible that one's experience with the vaccine might later inform their attitudes. However, a recent analysis of antivaccination attitudes in 24 countries suggests an interesting model for understanding the roots of such beliefs [34] which, in the case of vaccines, are ‘conspiratorial beliefs, reactance and disgust/fear towards blood and needles’ [21].

We found that the six items intended to measure attitudes towards vaccines adequately converged into one factor. Our means of measuring Vaccine Attitudes expressed positions towards vaccines’ efficacy and safety with those having more negative attitudes more frequently agreeing that ‘People get sick from vaccines’ and that ‘Vaccines weaken the immune system’. We also found that those with heightened Disgust Sensitivity or Germ Aversion tended to hold more negative attitudes towards vaccines. Prior research has found that adults scoring higher on disgust sensitivity were more likely to adhere to the misconception that vaccines cause autism and were more skeptical of the effectiveness and safety of vaccines [19]. In another study, greater disgust sensitivity was associated with antivaccination beliefs, support for organic foods and opposition to genetically modified foods [20]. A 2018 study of 24 countries found that those with antivaccination attitudes scored higher on conspiratorial theory, reactance and disgust towards blood and needles [21]. These findings suggest that attitudes towards vaccination may be less subject to rational thinking and more driven on an emotional basis and, in particular, disgust. Accordingly, our mediation analyses’ results show that Disgust Sensitivity and Germ Aversion had a negative indirect effect on vaccine uptake which was mediated by Vaccine Attitudes. That is, heightened Disgust Sensitivity, when mediated by Vaccine Attitudes, leads to a decreased odds of vaccine uptake. This finding may help explain the contradictory findings observed among health care workers, a high-risk population with knowledge of disease transmission mechanisms and access to vaccines, who seldom achieve coverage targets without additional incentives [35] or resort to litigation when faced with mandated vaccination [13]. A qualitative review among health care workers in Europe suggested an emotional basis for decision making highlighting health care worker's concern with vaccine side effects as well as distrusts of pharmaceutical companies [36].

A recent ecological study evaluated the association between a population's cultural and social norms assessed via voting in the US 2012 presidential election with uptake of routine adolescent vaccines at the state level [37]. Those authors found that states classified as ‘red’ (i.e. with Republican affiliation) had significantly lower vaccine coverage of human papillomavirus (HPV), tetanus-containing (Tdap) and meningococcal (MCV4) vaccines. Political ideology and attitudes towards a diverse set of policies and social norms have been shown to correlate with disgust sensitivity in different cultures [14]. Though most studies have been conducted in countries where English is the primary language, a recent Dutch study corroborated the association of disgust with political ideology showing how heightened disgust sensitivity correlated with the socially conservative as well as with negative attitudes towards immigrants, gays and lesbians, and greater nativism and isolationism [38]. As such, disgust is involved in the avoidance of physical threats (pathogens) as well as perceived ones and vaccine attitudes may result from disgust's role in an individual's political, social and moral beliefs. In a study of prejudice against immigrants and foreigners, authors found the link between disgust sensitivity and prejudice was not due to concerns about disease acquisition but by ideological orientation [39].

Notwithstanding, we also found that Disgust Sensitivity and Germ Aversion had a direct positive effect on vaccine uptake, i.e. those with heightened disgust sensitivity were more likely to get vaccinated. Indeed, as suggested in the introduction, disgust may have two opposing associations with vaccine uptake. Since vaccines prevent infection, those with heightened disgust sensitivity might vaccinate more; this likely reflects a rational decision-making process (our direct positive effect). Our results also show that those who perceive themselves more susceptible to infectious diseases, that is, who scored higher in Perceived Susceptibility, were significantly more likely to report following through on the expected manifest behaviour, that is, getting vaccinated. A 2017 systematic review that included 470 studies reported similar findings: low perceived risk of acquiring the disease was a significant barrier to vaccine uptake [5]. Interestingly, we found no indirect effect (be it positive or negative) of Perceived Susceptibility on vaccine uptake that could be mediated by Vaccine Attitudes.

We also found that the effects of Disgust Sensitivity, Germ Aversion and Perceived Susceptibility were no longer statistically significant when we included in the models other covariates such as workplace availability of the vaccine, belonging to a priority group and perceived barriers of flu vaccine [23]. These results suggest that although heightened disgust sensitivity may correlate with negative attitudes towards vaccines, as suggested by us and others [19, 20], its impact on vaccine uptake is minimal. However, it is important to note that our inclusion of these variables as covariates in the regression models imply a specific causal structure where these covariates are interpreted as ‘confounders’ and the regression coefficient the ‘adjusted effect’ of the treatment variable. It may well be that other theoretical models could consider the interplay of these factors in different manners, thus highlighting a possible effect that was obscured in this analysis. Indeed, this is what we see in the unadjusted effects of Disgust Sensitivity and Germ Aversion: had we only considered their total effect, we would have concluded that there was none. However, when we separated their effects into a direct effect on the outcome and an indirect effect mediated by Vaccine Attitudes, we observed the two opposing effects.

Moreover, the lack of effect of Disgust Sensitivity on reported flu vaccine uptake in the model that considered other covariates may be due to our assessment of the manifest behaviour as specific to the influenza vaccine, that is, disgust may impact vaccine uptake differently depending on the infection/vaccine [40]. However, a 2017 systematic review of 145 European studies on vaccine perceptions and concerns for various vaccines (seasonal and pandemic influenza, HPV, MMR, others) found that safety, in particular, mistrust, was the largest deterrent to vaccine uptake in all countries, for all vaccines [41].

Some of the limitations of the present study have been discussed previously [23] and include the small, non-probabilistic nature of the participant selection, the MTurk workforce [25]. Moreover, as with any survey, the data are self-reported and not verified, however, the MTurk population has been shown to be at least as honest and reliable as other survey respondents [25]. As for external validity, our subjects were younger and with higher income and education that the general US population. In the present, though we used two validated instruments to measure Disgust Sensitivity, Germ Aversion and Perceived Susceptibility, we highlight that others are available such as the Three-Domain Disgust Scale [42] and that future studies could address the roles the different domains of disgust might have. Finally, we highlight that our six-item Vaccine Attitudes measure, though adequately consistent, was not validated and suggest, for future studies, the currently available measures of vaccine hesitancy particularly the 5C scale (Confidence, Constraints, Complacency, Calculation and Collective) given its comprehensiveness in considering all psychological antecedents of vaccination, both individual and social [43].

As suggested in multiple studies, a natural and necessary next step is the development of means of communicating with individuals to promote acceptance, uptake and adherence to vaccine recommendations. Our findings suggest disgust might drive negative attitudes towards vaccines though to improve flu vaccine uptake it would be more effective to address economic, logistic and psychological barriers. A 2017 review that integrates diverse findings from psychology, sociology, behavioural economics, public health and medicine suggests a similar path, that which acknowledges and includes psychological theories to promote vaccine uptake [44]. Prior trials unfortunately show how tricky message framing for vaccine uptake can be. A 2014 randomised online trial tested four message framing interventions, none of which increased intention to vaccinate [18]. Two 2015 studies found that correcting myths about vaccine's side effects (i.e. flu vaccine causes the flu [10] and measles, mumps and rubella (MMR) vaccine causes autism [12]) had no effect on vaccine intention or attitude, respectively. A 2017 study on the effect of self-affirmation exercises found it to be ineffective or even detrimental when correcting information regarding vaccine safety was not jointly provided [11]. To this effect, recent work by Hornsey *et al.* [21, 34] shed new light into the roots of antivaccination attitudes while also proposing a new model for promoting vaccine acceptance that seems promising.
